# Microbial Life in Playa-Lake Sediments: Adapted Structure, Plastic Function to Extreme Water Activity Variations

**DOI:** 10.1007/s00248-024-02454-4

**Published:** 2024-11-09

**Authors:** Judit Boadella, Andrea Butturini, Anna Doménech-Pascual, Zeus Freixinos, Núria Perujo, Jordi Urmeneta, Ariadna Vidal, Anna M. Romaní

**Affiliations:** 1 GRECO, Institute of Aquatic Ecology, University of Girona, Av. Mª Aurèlia Capmany, 69, 17003 Girona, Spain; 2 Department of Evolutionary Biology, Ecology and Environmental Sciences, University of Barcelona, Av. Diagonal 643, 08028 Barcelona, Spain; 3 Department of Ecology and Hydrology, Faculty of Biology, University of Murcia, Campus de Espinardo, 30100 Murcia, Spain; 4 Department of River Ecology, Helmholtz Centre for Environmental Research – UFZ, Brückstraße 3a, 39114 Magdeburg, Germany; 5 Department of Genetics, Microbiology and Statistics, University of Barcelona, Diagonal 643, 08028 Barcelona, Catalonia Spain; 6 Biodiversity Research Institute, University of Barcelona, Diagonal 643, 08028 Barcelona, Catalonia Spain

**Keywords:** Hypersaline, Drought, Biofilm, Water activity, Enzyme activity

## Abstract

Saline shallow lakes in arid and semi-arid regions frequently undergo drying episodes, leading to significant variations in salinity and water availability. Research on the impacts of salinity and drought on the structure and function of biofilms in hypersaline shallow lakes is limited. This study aimed to understand the potential changes of biofilms in playa-lake sediments during the drying process. Sediments were sampled at different depths (surface, subsurface) and hydrological periods (wet, retraction, and dry), which included a decrease in water activity (a_w_, the availability of water for microbial use) from 0.99 to 0.72. a_w_ reduction caused a greater effect on functional variables compared to structural variables, indicating the high resistance of the studied biofilms to changes in salinity and water availability. Respiration and hydrolytic extracellular enzyme activities exhibited higher values under high a_w_, while phenol oxidase activity and prokaryote biomass increased at lower a_w_. This shift occurred at both depths but was more pronounced at the surface, possibly due to the more extreme conditions (up to 0.7 a_w_). The increased levels of extracellular polymeric substances and carotenoids developed at low a_w_ may help protect microorganisms in high salinity and drought environments. However, these harsh conditions may interfere with the activity of hydrolytic enzymes and their producers, while promoting the growth of resistant prokaryotes and their capacity to obtain C and N sources from recalcitrant compounds. The resilience of biofilms in hypersaline lakes under extreme conditions is given by their resistant biochemichal structure and the adaptability of their microbial functioning.

**Supplementary Information:**

The online version contains supplementary material available at 10.1007/s00248-024-02454-4.

## Introduction

Saline lakes are mostly found in endorheic basins in semi-arid and sub-humid climates in which evaporation exceeds precipitation. It is common for saline shallow lakes to contain water only intermittently, and under these conditions, the variability of water volume is associated with changes in salinity [1]. In hypersaline lakes, changes in both salinity and water content make the availability of water for microbial use (water activity, a_w_) scarce, which may compromise organisms if they are not well-adapted [2, 3]. It has been described that microbial life (archaea, bacteria, and fungi) has a limit for biotic activity at 0.585 a_w_ [4].

In sediments, most biomass can be found at the surface, organized in highly structured biofilms as microbial mats or less structured consortia of organisms adhered to sediment particles [5]. In biofilms, autotrophs and heterotrophs are embedded in an extracellular polymeric substances (EPS) matrix, which is composed of a biopolymer mixture (mainly polysaccharides and proteins) and provides cohesion to the biofilm. The EPS also gives resistance to environmental stress factors, helps to retain extracellular enzymes, and makes surface sediments a hot spot for microbial activity [6–8]. Adaptations of sediment microorganisms to water scarcity and high salinity include the accumulation of osmolytes or the development of a thick matrix of EPS [8, 9]. Though some adaptations to both salinity and drought may be similar (e.g. increased EPS matrix), microorganisms have different resistance to these stressors, and so, depending on the specific stress, the biochemical structure (henceforth referred to as “structure”) of the biofilm could change in a different direction. For example, some studies suggest that fungi may exhibit greater resistance to drought than bacteria [10, 11], while bacteria may demonstrate higher tolerance to salinity than fungi [12, 13]. In this context, archaea are recognized for their high resistance to both drought and salinity [14, 15]. A biofilm’s structure can also be affected by changes in primary producers, which have been reported to be negatively affected by salinity [16] and drought [17], although primary producers may adapt to such harsh conditions through accumulation of carotenoids or lipids [18, 19]. Furthermore, specific changes in lipid composition have been related to metabolic stress of the microbial community, as the *trans/cis* ratio of phospholipid fatty acid (PLFA) reflects the response of gram-negative bacteria to environmental stresses [20, 21].

Suffering from salinity and drought stress exposure can change not only the structure of a biofilm, but also its functionality or activity. Respiration and extracellular enzyme activity measures provide a meaningful view of the activity of a biofilm in relation to the use of organic matter [22, 23]. Salinity and drought have been observed to cause an inhibition on respiration and extracellular enzyme activities in wetlands [24–26], although they may enhance oxidative carbon-acquiring enzymes in saline wetlands [27].

Sediment depth can also play a role in the microbial responses of ecosystems that suffer from harsh conditions, since subsurface layers can act as a refuge. It has been seen in a hypersaline shallow lake similar to our study site that subsurface sediments have higher enzyme efficiency than surface sediments, and this has been related to a multiple stressors (sun radiation and salinity) present in the surface and not in the subsurface [28]. Similarly, from intermittent river ecosystems, it has been described that subsurface sediment kept moisture content and extracellular enzyme activities [29], and was much less sensitive to long drought episodes than the surface sediment [30].

It is still not clear how the availability of water can affect the structure and the functionality of the biofilm in sediments of hypersaline lakes where drought and salinity co-occur and reach extreme values. Water activity (a_w_) is used here as a measure that integrates both water scarcity due to drought and low water availability due to hyper-salinity, and is directly related to limits for life development.

The main objective of this study was to determine how water activity affects the functioning and the structure of the sediment’s biofilm in an environment that naturally suffers from huge variability in water availability, and if the effect was the same in two different sediment depths. The term “structure” throughout the manuscript refers to the biochemical structure or chemical composition, as a reflection of the distinct microbial biomass (e.g. prokaryotes) and the development or secretion of resistance strategies (e.g. EPS). To this aim, surface and subsurface sediment were collected from a playa-lake at three different hydrological periods (wet, water retracted, and dry) for the analysis of heterotrophic microbial metabolism (respiration and extracellular enzyme activities), and structural properties (content of protein, lipid, EPS, pigments, and biomass of prokaryotes and fungi) (Suppl. Fig. 1). It was expected that the driest conditions would cause a reduction of the biofilm’s functioning, and also induce changes in the biofilm’s structure by increasing those structures that help resist harsh conditions (EPS and carotenoid content) and the biomass of the most persistent microbial group. Furthermore, it was hypothesized that changes would be greater at surface than subsurface due to its higher salt accumulation during drought.

This paper provides new insights into the impact of salinity and drought on biofilms in hypersaline shallow lakes, and it offers a perspective on both their functionality and structure through a wide range of biochemical analyses. Additionally, it introduces the use of a_w_ as a sediment measure that integrates the effects of both salinity and drought.

## Methods

### Study Site

This study was performed at the *Salada de La Muerte* shallow saline lake. La Muerte is an endorheic temporary playa-lake located in the Ebro depression (Monegros district, NE Spain), an area with a mean annual precipitation of 350 mm [5] (Suppl. Fig. 2). The average annual temperature in the area for 2020 was 14.1 °C. Monthly mean temperatures were 7 °C in January–February (wet sampling), 21.2 °C in June (retraction sampling), and 23 °C in July (dry sampling). Additionally, temperatures were highly variable daily and monthly, with the minimum/maximum temperatures of − 1.2/21.6 °C in February, of 8/35.1 °C in June, and 10.4/37.2 °C in July (at Bujaraloz municipality at the Monegros area, 17 km from La Muerte playa-lake, data from IAEST, institute of statistics from the Aragón region government). The surface area of the playa-lake is 0.18 km^2^, but only 0.08 km^2^ are usually flooded [31]. Due to its temporary nature, this playa-lake has a wide range of salinity, with conductivities varying from 20 to 130 mS cm^−1^, depending on the water column depth. La Muerte sediment particles are mainly medium (0.25–0.5 mm) and coarse sand (0.5–2 mm), which allows the development of microbial mats [32, 33]. The biofilm at La Muerte maintains a thickness of less than 2 mm throughout the year. When the playa-lake has surface water, it forms a microbial mat easily identifiable and separable from the sediment. Conversely, during dry periods, the biofilm appears more adhered and mixed with the sediment particles and exhibits a visually drier appearance.

### Sampling Design

Sampling was performed during 2020 in three moments related to three different hydrological periods: wet, water retracted (henceforth named “retraction period”), and dry. Sampling for the wet period was performed on 28 January when the playa-lake had surface water (approximately 0.08 km^2^ of surface water and 13.5 cm water’s depth). Retraction period sampling was performed on 15 June when the playa-lake was drying up (approximately 0.02 km^2^ of surface water and less than 1-cm water depth). The dry period sampling was performed on 20 July, when the playa-lake was completely dry. At each sampling period, five replicate sediment cores were collected from the playa-lake. Replicate cores were taken 20 m apart from each other, and the area where each replicate was collected coincided between sampling periods. Cores had a diameter of 10 cm and were 15-cm deep. Cores were transported to the laboratory and kept at 4 °C. The day after sampling, 1 cm^3^ subsamples were separated from each core by using an uncapped syringe distinguishing two depths: surface (consisting of the first cm depth from the sediment surface, including the surficial microbial mat/biofilm), and subsurface (consisting of samples from the deepest part of the core, around 15-cm depth from the surface sediment; approx. 12- to 15-cm depth, Suppl. Fig. 1).

On the wet sampling, 2 l of water from La Muerte playa-lake was sampled to be later used for the extracellular enzyme activities and aerobic respiration incubations. The water was transported to the laboratory and kept at 4 °C until the next day, when it was filtered with a 0.2-µm filter (nylon filter, 0.2-µm pore size, Whatman International Ltd.). Filtered water was stored at 4 °C and was kept in order to be used for all the incubations (i.e. wet, retraction, and dry sampling incubations).

At the wet and retraction samplings, the physicochemistry of water (pH, salinity, redox potential, temperature, and conductivity) was measured by using portable metres (Sension + PH1, Hach Lange; HRS”8-T, Kruss; WTW pH 340i, ICT; HI-98192 EC/TDS/Resistivity/Salinity, Hanna Instruments). Water samples were also collected and filtered (0.2-µm nylon filters) to measure nitrate (Bran + Luebbe autoanalyser equipped with a reductive copper cadmium column) and soluble reactive phosphorus content (ammonium molybdate colorimetric method, following Murphy and Riley [34]), and acidified to measure the dissolved organic carbon (DOC) using combustion catalytic oxidation (TOC-VCSH Analyser, Shimadzu, Kyoto, Japan). Water DOC content was measured in wet and retraction periods while water nutrients were only measured in the wet period.

### Water Availability

Water availability in the playa-lake sediments was measured as water activity and as water content. For all samplings, water activity at the surface and subsurface sediment was measured for each replicate with the a_w_ metre LabSwift-aw (Novasina AG, Lachen, Switzerland), as an indicator of water available for microbial activity [3].

Water content was measured from the 1 cm^3^ samples by first weighing the fresh samples, drying them (70 °C, until constant weight, ca. from 48 to 96 h), and weighing to obtain the dry weight (DW). Water content is expressed as a percentage from (fresh weight – DW) · fresh weight^−1^.

### Structural Characterisation of the Playa-Lake Sediment

#### Organic Matter and Carbon Content

To measure the total organic matter content, dried samples (those used for water content analysis) were then burnt for 4.5 h at 450 °C (MF12-124, Hobersal, Barcelona, Spain), and weighted again. Organic matter results are expressed as grammes of ash-free dry weight (AFDW) per gramme of DW. All variables in this study have been standardized by DW.

Carbon content was measured with an elemental analyser (CE EA 1108, Thermo Fisher Scientific) using sulphanilamide as the standard.

#### Protein, Lipid, and Polysaccharide Content

##### Total Protein Content

Protein content in sediment samples (1 cm^3^) was determined following Lowry et al. [35] modified by Rice [36] to compensate phenol interference. Samples for protein analysis were kept frozen (− 20 °C) until analysis. An extraction with near-boiling NaOH (1N, 10 min) was performed before the analysis. The extracts were then cooled and centrifuged (2000 g, 2 min). Supernatant was separated into two aliquots of extract. An alkaline copper solution was added to the first extract, while an alkaline reagent without cupric sulphate was added to the second aliquot [36]. The mixture was left to react for 10 min (dark conditions) and 1N Folin reagent was added. Mixtures were left to stand for 30 min at dark to let the colour develop before measuring the absorbance at 750 nm. The difference between aliquots containing or not cupric sulphate was performed, and protein quantification was done by using bovine serum albumin as standard (0–100 mg L^−1^). Results are given as mg of proteins g DW^−1^ of sediment.

##### Phospholipid Fatty Acid Analysis (PLFA)

The original protocol by White [37] was followed with modifications. Freeze-dried samples (1.5 to 3 g) were pulverized and total lipids extracted. The phospholipid fatty acids were transesterified into methyl esters with KOH and identified by gas chromatography/mass spectrometry (GC/MS). PLFA are products of biosynthetic pathways and reflect phenotypic responses of microorganisms to environmental conditions and thus indicating the nutritional and physiological status/stress of the microbial community. Here, the trans/cis ratio ((16:1*ω*7t / 16:1*ω*7c) + (18:1*ω*7t / 18:1*ω*7c)) and the cyclo/monoenoic ratio ((cy17 / 16:1*ω*7c) + (cy 19 / 18:1*ω*7c)) were calculated to assess the metabolic stress of the gram-negative bacterial community. Trans/cis ratio is associated with starvation and cyclo/monoenoic ratio is associated with slow growth [20, 21]. Furthermore, total PLFA provides a quantitative measure of the viable or potentially viable microbial biomass.

##### Polysaccharide Content in Extracellular Polymeric Substances (EPS)

Samples used to measure polysaccharide content in EPS were kept frozen (1 cm^3^ samples, − 20 °C) until analysis. EPS was extracted from the sediment using a cation exchange resin (Amberlite HPR1100, Sigma-Aldrich, Merck KGaA, Darmstadt, Germany) by following Romaní et al. [38]. Following the extraction, polysaccharide content in EPS was quantified by the phenol–sulphuric acid method [39]. A standard curve of glucose (0–100 µg mL^−1^) was used to quantify the polysaccharide content. Results are expressed as µg of glucose-equivalent g DW^−1^ of sediment.

#### Microbial Biomass and Pigment Content

##### Prokaryote Density

Prokaryote density from the sediment samples was measured following Amalfitano et al. [40]. Sediment samples (1 cm^3^) were fixed with a detaching solution that contained formaldehyde [41] and were kept at 4 °C until analysis. On the day of the analysis, samples were shaken (30 min) and sonicated (2 cycles of 1 min). Nycodenz (optiprep density gradient, Sigma-Aldrich, Merck KGaA, Darmstadt, Germany) was added to the samples and then samples were centrifuged (90 min, 14,000 g, 4 °C). The supernatant was stained with Syto13 (5 µM, Thermo Fisher Scientific, Waltham, MA, USA) and a beads solution was added as an internal pattern. When necessary, samples were diluted with sterilized Ringer solution before staining. Prokaryote density was determined by flow cytometry (FACSCalibur, Becton Dickinson, Franklin Lakes, NJ, USA). Results are given as cells g DW^−1^ of sediment.

##### Fungal Biomass

Fungal biomass was determined from ergosterol analysis by following Gessner and Schmitt [42]. Sediment samples (1 cm^3^) were kept frozen (− 20 °C) until their analysis. Samples were lyophilized and incubated with 0.14 KOH in methanol (30 min, 80 °C, shaking) to extract lipids from the sediment. The obtained extracts were then filtered (glass fibre filters GF/C, 1.2-µm pore size, Whatman International Ltd.) and ergosterol was separated from the extracts by solid-phase extraction (Sep-Pak tC18 6 cc Vac Cartridge, 500 mg, Waters Corporation, Milford, MA, USA). Ergosterol was quantified by high-pressure liquid chromatography (HPLC analyser, Waters Corporation) using the Nova-Pak C18 4 µm 3.9 × 300 mm column (Waters). Ergosterol was quantified using ergosterol standards (0–200 mg L^−1^). A conversion of ergosterol to fungal biomass was made by using the conversion factor of 5.5 mg of ergosterol in 1 g of fungal biomass [43] and 43% of fungal C in fungal dry mass [44]. Results are expressed as µg C g DW^−1^ of sediment.

##### Pigment Content

Samples used to analyse pigment content were kept frozen (− 20 °C) until analysis. Pigments were extracted from the sediment following Jeffrey and Humphrey [45] by using 90% acetone (24-h incubation in cold for passive extraction). Samples were sonicated (sonication bath Ultrasons, Selecta) and filtered (glass fibre filters GF/C, 1.2-µm pore size, Whatman International Ltd.) and the absorbance spectrum was measured at 200–800 nm with 1-nm intervals (UV-1800, Shidmadzu). Algorithms by Ritchie [46] were used to quantify chlorophyll-*a* and bacteriochlorophyll-*a*, and algorithms by Strickland and Parsons [47] modified by Jodłowska and Latała [48] were used to determine carotenoid content. Pigment content results are expressed as µg pigment g DW^−1^ of sediment. Margalef index (OD 430/665) was also calculated as the ratio Abs430/Abs665 as an indicator of the proportion of degraded chlorophyll and protection pigments such as carotenoids, to active chlorophyll [49].

### Functional Characterisation of the Playa-Lake Sediment

#### Aerobic Microbial Respiration (Resazurin Consumption)

Aerobic respiration was assessed by measuring the consumption of resazurin [50]. Incubations to analyse the aerobic respiration were performed with fresh samples, i.e. samples kept at 4 °C until the analysis and analysed less than 24 h after the sampling moment. Resazurin was added to the 1 cm^3^ sediment samples along with La Muerte filtered water at a final concentration of 60 µM. Incubations lasted 2 h in agitation, 20 °C, and darkness conditions. A control with filtered water and resazurin (without sample), a blank with filtered water and sample (without resazurin), and quenching effect (interaction between resorufin and sediment that could affect the measured absorbance) were measured. After the incubation, samples were centrifuged (2 min, 2000 g) and the absorbance was measured at 603 nm (Infinite M200 PRO, Tecan, Zurich, Switzerland). Quantification of resazurin consumption was performed using a resazurin standard (0–250 µM). Results are expressed as µmols of resazurin consumed g DW^−1^ of sediment per hour.

#### Extracellular Enzyme Activities

Extracellular enzyme activities were measured with fresh samples (1 cm^3^) less than 24 h after the sampling. Samples were kept at 4 °C until the analysis. Phenol oxidase and six hydrolytic enzyme activities were measured (Table 1). All incubations were performed under substrate-saturating conditions. Saturation curves with samples from the study site were performed before this study to determine the concentration of each enzyme substrate during the incubations. The chosen concentrations were maintained during the three sampling periods.

**Table 1:** Summary of the extracellular enzyme activities used in this study, the artificial substrate used to measure each enzyme activity, and its saturation concentration. Degradation roles obtained from Romaní et al. [51]. *MUF*, methylumbelliferyl; *AMC*, amido-4-methylcoumarin

Enzyme (abbreviation)	Enzyme commission number	Degradation role	Artificial substrate	Saturation concentration (µM)
*β*-glucosidase (GLU)	EC 3.2.1.21	Cellulose (cellobiose, simple polysaccharides)	4-MUF *β*-glucopyranoside	0.3
*β*-xylosidase (XYL)	EC 3.2.1.37	Hemicellulose (xylobiose, simple polysaccharides)	4-MUF *β*-D-xylopyranoside	0.8
Cellobiohydrolase (CBH)	EC 3.2.1.91	Cellulose	4-MUF *β*-D-cellobioside	0.8
Leu-aminopeptidase (LAP)	EC 3.4.11.1	Peptide degradation (split leucine amino acid from a peptide chain)	L-leucine-7-AMC hydrochloride	0.3
Phosphatase (AP)	EC 3.1.3.1–2	Phosphomonoesters	4-MUF phosphate	0.3
Sulfatase (SUL)	EC 3.1.6.1	Aryl sulphate	4-MUF sulphate potassium salt	0.8
Phenol oxidase (PO)	EC 1.14.18.1	Lignin	L-DOPA (L-3,4-dihydroxyphenylalanine)	1500

Phenol oxidase was measured using the L-dihydroxyphenilalanine (L-DOPA) substrate by following Sinsabaugh et al. [52]. Incubation was performed with acetate buffer and La Muerte filtered water (1:1 volume:volume) and L-DOPA final concentration of 1.5 mM on agitation and in darkness (2 h, 20 °C). Controls of the samples without L-DOPA were incubated following the same procedure. After the incubation, absorbance at 460 nm was measured (Infinite M200 PRO, Tecan). Results are expressed as nmols of DIQC g DW^−1^ of sediment per hour.

For the measurement of hydrolytic extracellular enzyme activities, sediment samples incubations were performed with La Muerte filtered water (0.2-µm nylon filters) and each artificial substrate added to reach saturation conditions (Table 1), on agitation and in darkness (1 h, 20 °C). A blank for each substrate was incubated also with filtered water (without sediment sample), controls of each sample were incubated with filtered water (without artificial substrate), and the quenching effect (potential interaction between MUF or AMC and sediment which could cause fluorescence to be underestimated) was also measured for surface and subsurface sediments [53]. Glycine buffer (pH 10.4) was added to stop the reaction, and then samples were centrifuged (2 min, 2000 g). Fluorescence of the supernatant was measured in a 96-well black plate. Samples incubated with MUF substrates were measured at excitation/emission of 365/455-nm wavelengths, and those incubated with AMC substrates at 364/445-nm wavelengths using a microplate reader (Infinite M200 PRO, Tecan). Enzyme activity was quantified by preparing MUF and AMC standards (0–100 µM). Results are expressed as nmol of MUF or AMC released g DW^−1^ of sediment per hour.

### Statistical Analyses

All statistical analysis was performed with R, using the base R package and the *ggplot2*, *FactoMineR*, and *factoextra* packages.

An analysis of covariance (ANCOVA) was applied to all the structural and functional variables with the water activity (a_w_) measured for each replicate used as a covariable, and the depth as a factor (surface vs. subsurface), in order to test for the effect of a_w_ and depth and to see whether effect of a_w_ was being different in surface than in subsurface. Normality and homoscedasticity were checked before applying the ANCOVA, and variables were transformed when necessary (water content, carbon content, EPS, total proteins, PLFA, metabolic stress indices, prokaryote density, chlorophyll-*a*, carotenoid, bacteriochlorophyll-*a*, Margalef index, *β*-glucosidase, *β*-xylosidase, cellobiohydrolase, leu-aminopeptidase, phosphatase, and phenol oxidase needed to be transformed by logarithm).

For the comparison of the biological characterisation among depths, the relative contribution of each compartment (EPS, prokaryotes, algae, fungi) was calculated from values expressed in µg C g DW^−1^ and averaged for each sampling period. Prokaryote densities were transformed into C by using conversion factors [54] and considering a mean cell biovolume of 0.29 µm^3^ [55]. Chlorophyll-*a* was transformed into C following Geider and MacIntyre [56]. Values from prokaryotes and fungi expressed in µg C g DW^−1^ were also used to calculate the prokaryote/fungi ratio. The use of these fixed conversion factors to transform values into carbon units has certain limitations but allows to obtain a proxy for the comparison of the relative biomass of the different groups in depth and depending on water availability. This comparison might be slightly biased because the measurement of prokaryote density includes cyanobacteria, which contribute to autotrophs and are included in the chlorophyll content analysis.

Principal component analysis (PCA) using all variables was performed in order to see the relationships between structural and functional variables in the studied sediment and at the different hydrological conditions. Data used for the PCA was standardized, and a Cos2 of 0.5 was applied to the graph to discard those variables with lower quality.

## Results

### Water Physicochemistry of La Muerte Playa-Lake

Table 2 summarizes the physicochemical parameters of La Muerte, indicating large differences between the wet and the retraction periods, such as the increase in conductivity, salinity, and DOC content, and decrease in redox potential.

**Table 2:** Physicochemical parameters measured in La Muerte surface water for wet and retraction sampling periods. No data are shown for dry period since there was no surface water available in the shallow lake. *NA*, not available

	Wet	Retraction
Temperature (°C)	10.0	32.0
Conductivity (mS cm^−1^)	24.8	450.0
pH	8.32	9.6
Salinity (%)	2.2	25.5
Redox potential (mV)	337.0	125.0
DOC (mg L^−1^)	6.34	156.38
NO_3_^−^ (mg L^−1^)	0.032	NA
PO_4_ (mg L^−1^)	0.041	NA

### Water Availability of La Muerte Playa-Lake Sediment

Sediment water content was higher at the surface than at the subsurface, while no significant differences in a_w_ between depths were found (Table 3). The range of a_w_ covered in the study was of 0.72–0.99 at the surface sediment and of 0.86–0.94 at the subsurface sediment. At the surface sediment, the relationship between water content and water activity was not linear; in the wet period, a_w_ greatly increased while water content was maintained in similar values to those in the retraction period. At the subsurface sediment the range for both a_w_ and water content was narrow, and in the retraction and dry periods, a_w_ was higher than those in the surface sediment, although water content was similar suggesting lower salinity (Suppl. Fig. 3).

**Table 3:** Water availability and structural variables measured in La Muerte for surface and subsurface sediments. Values are expressed as mean and standard deviation, including all collected samples from the three sampling periods (wet, retraction, and dry) (*n* = 15)

Variable	Surface	Subsurface
Water activity	0.82 ± 0.11	0.9 ± 0.02
Water content (% g water g FW^−1^)	36.25 ± 5.84*	30.63 ± 1.71
Organic matter (g AFDW g DW^−1^)	0.12 ± 0.02*	0.09 ± 0.02
Carbon content (%)	1.56 ± 0.46*	0.65 ± 0.08
Total protein content (mg g DW^−1^)	0.82 ± 0.37*	0.15 ± 0.11
PLFA content (mg g DW^−1^)	0.04 ± 0.02*	0.01 ± 0
Trans/cis ratio (starvation)	0.07 ± 0.06	0.08 ± 0.06
Cyclo/mono ratio (slowed growth)	0.66 ± 0.27	1.92 ± 0.69*
EPS (µg glucose-eq g DW^−1^)	191.96 ± 174.87*	10.01 ± 8.03
Prokaryote density (10^8^ cells g DW^−1^)	4.14 ± 3.59*	1.35 ± 1.09
Fungal biomass (µg C g DW^−1^)	205.65 ± 125.61	147.58 ± 83.32
Chlorophyll-*a* (µg g DW^−1^)	11.54 ± 6.23*	0.33 ± 0.21
Bacteriochlorophyll-*a* (µg g DW^−1^)	0.94 ± 0.61*	0.08 ± 0.06
Carotenoids (µg g DW^−1^)	16.68 ± 9.11*	0.92 ± 0.25
Margalef Index	5.07 ± 1.93	9.64 ± 4.26*
Carotenoids/chlorophyll-*a*	1.61 ± 0.82	5.28 ± 6.36*
Prokaryotes/fungi	0.09 ± 0.15*	0.04 ± 0.05

*Indicates significant higher values (*p* < 0.05) in surface or subsurface (see Table 4). The detailed ANCOVA analysis results for the relationship of each variable to water activity and interaction with depth are included in Table 4

### Structural Characterisation of La Muerte Playa-Lake Sediment

**Fig. 1:**
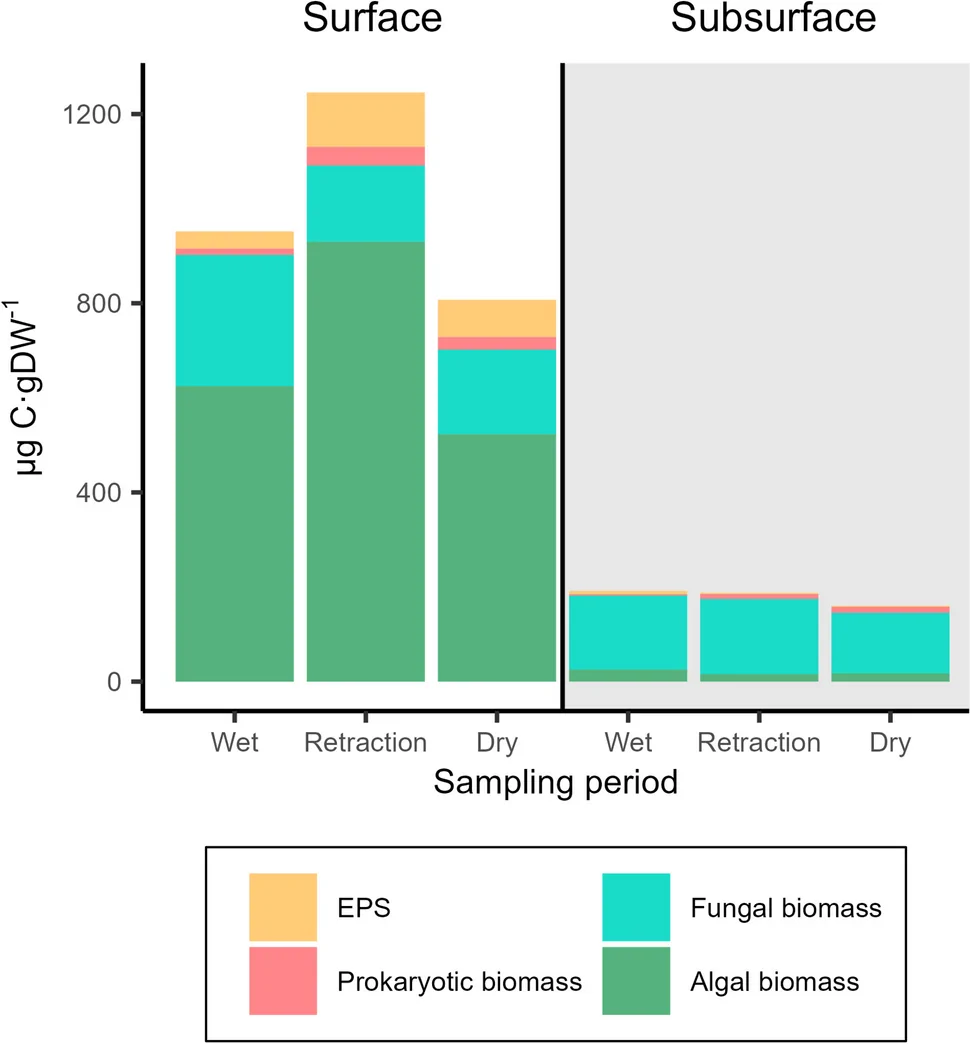
Content of extracellular polymeric substances (EPS), prokaryotes, fungi, and chlorophyll-*a* in the surface and subsurface biofilm (mean values from the five replicates in each depth and period). All values are expressed as µg of carbon g DW.^−1^

Contents of organic matter, carbon, total proteins, PLFA, polysaccharides in EPS, prokaryote density, and content of chlorophyll-*a*, bacteriochlorophyll-*a*, and carotenoids were higher in surface than in subsurface sediment. Fungal biomass was not significantly affected by depth but the prokaryote to fungi biomass ratio was higher in the surface sediment (Tables 3 and 4, and Fig. 1).

**Table 4:** *p*-values obtained from the ANCOVA results for structural variables and for extracellular enzyme activities and aerobic respiration. *Ns*, not significant

	Variable	Depth	a_w_	Depth:a_w_
Structural	Organic matter	** < 0.01**	Ns	** < 0.05**
	Carbon content	** < 0.0001**	Ns	Ns
	Total protein content	** < 0.0001**	Ns	Ns
	PLFA content	** < 0.0001**	** < 0.01**	Ns
	Trans/cis ratio (starvation)	Ns	Ns	Ns
	Cyclo/mono ratio (slowed growth)	** < 0.0001**	Ns	Ns
	EPS	** < 0.0001**	Ns	** < 0.01**
	Prokaryote density	** < 0.001**	** < 0.05**	Ns
	Fungal biomass	Ns	Ns	Ns
	Chlorophyll-*a*	** < 0.0001**	Ns	Ns
	Bacteriochlorophyll-*a*	** < 0.0001**	Ns	** < 0.05**
	Carotenoids	** < 0.0001**	Ns	Ns
	Margalef Index	** < 0.001**	Ns	Ns
	Carotenoids/chlorophyll-*a*	** < 0.001**	** < 0.05**	Ns
	Prokaryotic/fungal biomass	** < 0.05**	** < 0.01**	Ns
Functional	*Β*-glucosidase	** < 0.0001**	** < 0.01**	** < 0.05**
	*Β*-xylosidase	** < 0.0001**	** < 0.05**	Ns
	Cellobiohydrolase	** < 0.0001**	** < 0.001**	Ns
	Leu-Aminopeptidase	Ns	** < 0.001**	** < 0.01**
	Phosphatase	Ns	** < 0.01**	Ns
	Sulfatase	Ns	** < 0.0001**	** < 0.05**
	Phenol oxidase	Ns	** < 0.01**	Ns
	Aerobic respiration (resazurin)	** < 0.0001**	** < 0.001**	Ns

Contrarily, the cyclo/mono PLFA ratio, carotenoids/chlorophyll-*a* ratio, and Margalef index were higher in subsurface than surface sediments (Tables 3 and 4). The proportion of heterotrophic biomass (fungi and prokaryotes) was also higher in subsurface than in surface (0.93% and 1.70% for prokaryotes, and 20.89% and 84.73% for fungi, in surface and subsurface respectively), mainly due to the lower chlorophyll-*a* and EPS content in depth (Fig. 1).

Decreases in water activity affected positively PLFA content, prokaryote density, carotenoid/chlorophyll-*a*, and prokaryotic/fungal biomass ratio in both sediment depths, and also bacteriocholophyll-*a* content at the surface sediment. On the contrary, only at the subsurface sediment, a_w_ showed a positive relationship with EPS content and organic matter (ANCOVA, Tables 3 and 4, Suppl. Figure 4).

### Extracellular Enzyme Activities and Aerobic Respiration in La Muerte Playa-Lake Sediment

**Fig. 2:**
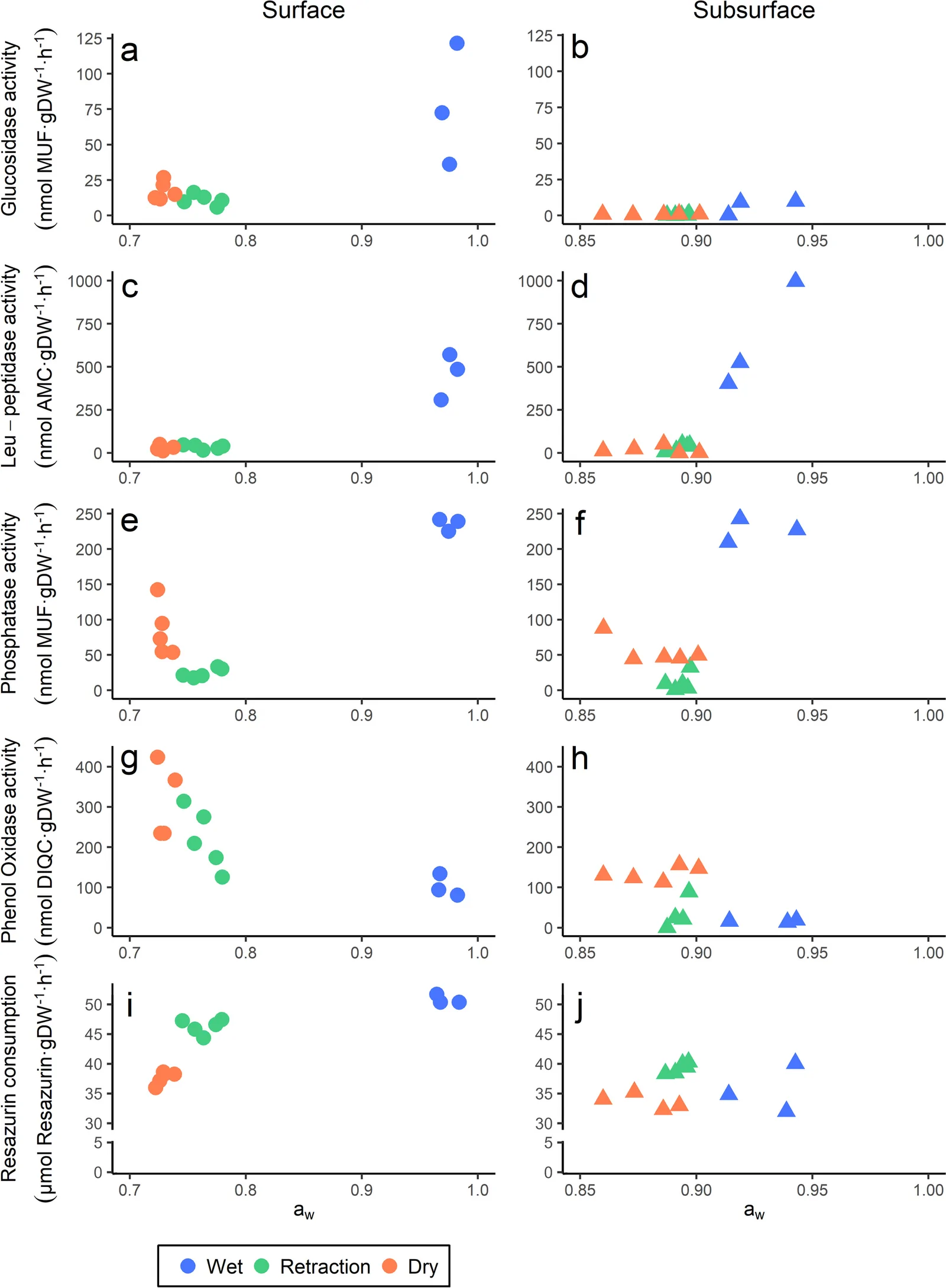
Extracellular enzyme activities and aerobic respiration measured in La Muerte for surface (left graphs) and subsurface (right graphs) sediments in the three sampling periods (wet, retraction, and dry). **a**, **b** *β*-glucosidase activity; **c**, **d** leucine-aminopeptidase activity; **e**, **f** phosphatase activity; **g**, **h** phenol oxidase activity; and **i**, **j** aerobic respiration as resazurin consumption

The extracellular enzyme activities involved in carbon use (*β*-glucosidase, *β*-xylosidase, and *β*-cellobiohydrolase), and the aerobic respiration were higher in surface than in subsurface sediments (Table 3, Fig. 2, Suppl. Figure 5), while all other activities measured did not show significant differences in depth.

All the activities measured in the studied playa-lake sediment showed a significant relationship with a_w_ but the sign of this relation was different depending on the specific activity and, for some activities, also depending on the depth (depth:a_w_ effects, Table 4). Hydrolytic enzyme activities and aerobic respiration showed a positive relationship with a_w_, in which the highest values were those with higher values of a_w_ (coinciding with those samples from the wet sampling period) (Fig. 2). Contrarily, phenol oxidase exhibited a negative relationship with a_w_, in which the maximum activity from this enzyme coincided with the lowest a_w_ (during the dry sampling period) (Fig. 2).

Three enzyme activities had depth:a_w_ effects. For *β*-glucosidase, the effect of a_w_ was higher in the surface, where differences between the wet period and retraction and dry periods were greater than in subsurface, while leu-aminopeptidase and sulfatase showed a greater effect of a_w_ in the subsurface (Table 4, Fig. 2, Suppl. Figure 5).

### Relationship Between Structural and Functional Variables in La Muerte Playa-Lake Sediment

**Fig. 3:**
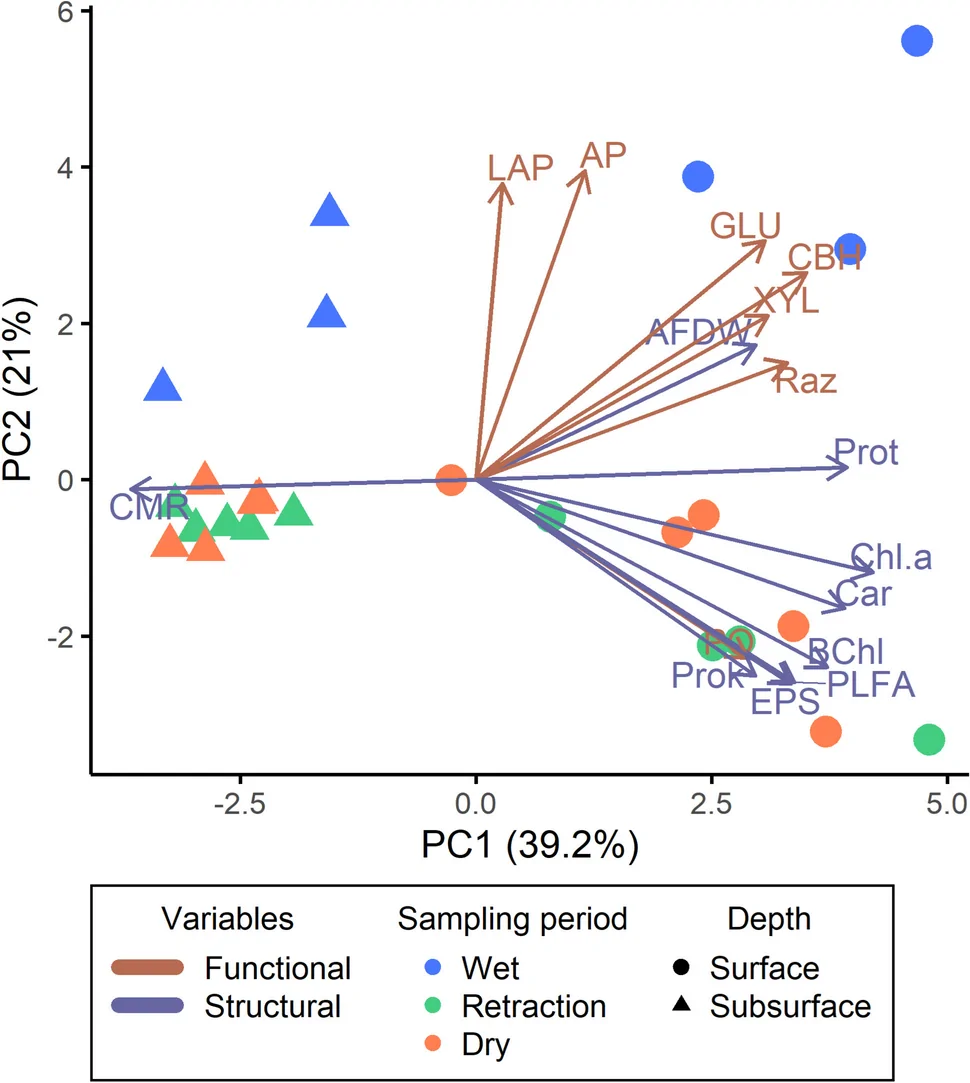
First two components of the PCA performed with all the structural and functional variables, including both sediment depths (surface and subsurface) and three sampling periods (wet, retraction, and dry). A Cos2 of 0.5 was applied to display those variables with stronger relationships with PC1 or PC2. AFDW, organic matter (ash free dry weight); EPS, extracellular polymeric substances; Prot, total protein content; PLFA, phospholipid fatty acid content; CMR, cyclo/mono ratio (slowed growth); Prok, prokaryote density; Chl.a, chlorophyll-*a*; BChl, bacteriochlorophyll-*a*; Car, carotenoids; GLU, *β*-glucosidase; XYL, *β*-xylosidase; CBH, cellobiohidrolase; LAP, leucine-aminopeptidase; AP, phosphatase; PO, phenol oxidase; Raz, aerobic respiration (resazurin)

The PCA incorporating all structural and functional variables clearly showed that both the depth of the sediment and the hydrological period (ca. sediment water activity conditions) influenced the sediment of La Muerte (Fig. 3). PC1 is related with the depth factor and differentiates surface samples on the right, from subsurface samples on the left. Most of the structural and functional variables have a positive relationship with PC1, indicating higher values at the surface than subsurface sediments. The cyclo/mono ratio shows a strong negative relationship with PC1, indicating that surface cells are closer to an exponential phase than subsurface cells (which are closer to a stationary phase, i.e. higher values of the index, as shown in Table 3). PC2 is associated with the hydrological period (ca. a_w_) showing the samples from the wet sampling period (with high a_w_) on top and samples from retraction and dry sampling periods (with low a_w_) on the bottom. Leu-aminopeptidase and phosphatase activities have a positive relationship with PC2, exhibiting higher activity at high a_w_ values (Table 4, Fig. 3). *Β*-glucosidase, *β*-xylosidase, *β*-cellobiohydrolase, organic matter, and aerobic respiration showed a strong relationship with those samples from surface sediments and high a_w_ values, while EPS, PLFA, prokaryote density, bacteriochlorophyll-*a*, phenol oxidase activity, and carotenoid content had a strong relationship with those samples from surface sediments and low a_w_ values (Fig. 3).

## Discussion

Microbial communities developing in shallow saline lakes are usually located at semi-arid or arid regions and suffer from several stress factors. Two of them—high salinity and dryness—are strongly linked to the availability of water. Liquid water availability has been clearly defined as a variable that determines the existence of life in any environment [57, 58]. For microbes, the limits for growth have been described at a water activity (a_w_) of 0.585, but most bacteria cannot grow below 0.9 a_w_ and most fungi are also inhibited below 0.8 a_w_ [3, 4]. Although in this study a_w_ did not reach the lowest extreme, a range between 0.72 up to 0.99 of a_w_ is included, with values of water content from 27.4 to 47.6%, and water salinity from 2.2 to 25.5%. This study shows discrete effects of a_w_ on structural parameters, but much generalized effects on functional parameters of the sediment microbial biofilm, with some effects differing between the surface and the subsurface, as discussed below.

### Little Sensitivity of Structural Parameters of Surface and Subsurface Sediments to Changes in a_w_

In the drying playa-lake sediments, the decrease in a_w_ caused an increase in the prokaryote biomass (as well as in PLFA content and the ratio prokaryotes/fungi) at both surface and subsurface sediments, with no changes to the other microbial compartment (fungi, algae) in terms of biomass (that was increasing from 7.6–17.3 and 1.4–4.9 µg C in wet to 9.6–90.4 and 2.8–24.4 µg C in drying and dry, in surface and subsurface respectively). This suggests that in such conditions of high salinity and low water content, and in both sediment depths, prokaryotes are more relevant for the sediment microbiology than fungi. We cannot say that fungi are inhibited since there is no significant effect of a_w_ in fungal biomass, but the greater contribution of prokaryotes than fungi in harsher conditions could be due to their better adaptation to a combination of high salinity and drought [12]. This could be further related to the growth of salt resistant bacteria, as described for soils [59]. Also, the lack of change in algal biomass due to the decrease in a_w_ suggests the community is already adapted mainly due to resistant strategies, especially at the surface sediment (as discussed below). It is important to note, in this context, that this was an in situ experiment, and it was not possible to control the temperature during the study. As a_w_ decreased, an increase in temperature was also observed. While the reduction in a_w_ may have affected microbial growth, temperature likely played a role as well. Specifically, bacteria may have been more favoured than fungi to grow under increasing temperatures [60, 61]. Nevertheless, a previous study in a playa-lake near La Muerte showed that day-night temperature fluctuations had little direct effects on microbial functioning, suggesting a great adaptation of these microbial communities to large temperature variations [28].

At the surface sediment, which reached lower a_w_ conditions (0.7) than the subsurface, further microbial structural responses were detected. The slightly increase in EPS (i.e. water stress and radiation resistance strategies), together with bacteriochlorophyll-*a*, suggests the growth of phototrophic bacteria, such as green or purple phototrophs. These organisms (known to have both carotenoid and bacteriochlorophyll pigments) usually grow in microbial mats below cyanobacteria, and some of them are well-adapted to extreme environments [62–64]. These responses only occurring at the surface sediment maybe related to the harsher a_w_ conditions but also solar radiation at the surface that confers a totally different structured sediment biofilm, with higher content of EPS and carotenoids than at the subsurface, as similarly found in a hypersaline shallow lake [28]. Also, at the surface sediment, high prokaryotes and primary producer biomass, together with their photosynthetic activity might be responsible for the greater accumulation of organic matter (as well as C and protein) than at the subsurface. Besides, at the subsurface, which reached less extreme conditions of water availability during the drying process and not suffering solar radiation, there was not any further structural effect than the increase in prokaryotes, and instead a_w_ reduction determined a slight decrease in EPS and organic matter content suggesting that the sediment’s capacity to retain organic matter was reduced with dryness.

### Water Activity Affects Surface and Subsurface Microbial Functioning in Hypersaline Shallow Lakes’ Sediment

In contrast to the structural parameters, functional differences between depths were less evident than the structural ones while microbial functions were highly sensitive to changes in a_w_. Overall, the main functional difference in depth was the greater enzyme capabilities for processing C compounds (*β*-glucosidase, *β*-xylosidase, *β*-cellobiohydrolase) and higher aerobic respiration at the surface than at the subsurface. This could be due to the organic matter that autotrophic organisms provide to heterotrophs [59] and the overall enhanced organic matter and C content, together with algal and prokaryotic biomass in the surface. However, all other measured enzymes involved in nutrient acquisition (leu-aminopeptidase and phosphatase), sulphur cycling, and lignin degradation (sulfatase, phenol oxidase) were equally active at both depths, suggesting that the subsurface sediment maintains organic matter cycling capabilities despite having lower microbial biomass. This could be due to the presence of active fungal and bacterial communities and/or high relevance of free extracellular enzymes at the subsurface. Furthermore, the cyclo/mono ratio from PLFA analyses indicates that subsurface microbes have a slower growth (stationary phase) than in the surface (closer to an exponential phase) [21, 65], which may suggest a potential lack of nutrients and readily degradable organic matter availability, prompting enzyme efficiencies [66].

Apart from the depth differences, a clear generalized effect of a_w_ was detected for all hydrolytic enzyme activities, which showed less activity associated with an increasing drought and salinity (a_w_ decrease). Lower extracellular enzyme activities cannot be explained by a reduction in the microbial biomass during drought, since an increase in prokaryote density was detected at lower a_w_, and no differences were detected for chlorophyll-*a* or fungal biomass. Though, even if functionality and structure showed different tendencies, aerobic respiration also exhibited a decrease along drought conditions, which suggest that maybe microorganisms were in a dormant state due to stress conditions [11, 67]. However, at the same time, phenol oxidase activity increased with lowest a_w_ at both sediment depth. Then, the functional change may be due to direct environmental effects and/or by indirect changes in the microbial community composition or the growth of resistant prokaryotes carrying other functions.

The effect of a_w_ on phenol oxidase activity is notable, exhibiting an increase with drought and salinity, contrary to hydrolases. This has been similarly observed with an increase in salinity in wetlands, which promotes microorganisms that synthesize oxidative enzymes as opposed to hydrolases [25]. Similarly, in drying streambeds, an increase in phenol oxidase when increasing drought intensity has been observed and related to a variation in the organic matter quality, shifting from a high availability of easy degradable compounds in wet conditions, to a greater accumulation of recalcitrant organic matter containing lignin in dry conditions [68]. In addition, drought likely increased sediment exposure to oxygen, which is required for phenol oxidase activity [69]. In peatlands, an increased aeration caused a rise in phenol oxidase activity, along with a decrease in hydrolytic activities [70]; and a rise in phenol oxidase activity under drought conditions was seen after an increase in bacterial growth rate [71]. This contrasts with the expectation that bacteria typically exhibit low phenol oxidase activity (i.e. degradation of complex compounds with aromatic rings), more associated with fungi [23, 72]. However, some studies have determined that bacteria can also produce phenol oxidase [73]. In the present study, prokaryote density has shown an increase with drought, and even if lack of data does not allow us to know which variable (prokaryote density or phenol oxidase activity) first responded to drought, there seems to be a clear relationship between them, and with drought.

## Conclusions

The structure of the playa-lake sediments was characterized by high organic matter, microbial biomass, respiration, and C-acquiring enzyme activity, together with high EPS and carotenoid content at the surface sediments. All variables decreased at the subsurface sediment except for nutrient acquiring enzyme activities that were maintained in depth. Most structural parameters showed no variability to changes in water activity, both in surface and subsurface sediments. Moreover, changes in the *trans*/*cis* ratio were not observed across the a_w_ gradient, but a mean value between 0.05 and 0.1 indicates that even if the community is suffering stress in some extent, it is not in starvation [21, 74]. This suggests a great adaptation of the community to the stress caused by high salinity and strong variations in a_w_ during drought and wet periods, since salt is always present in different amounts and drought is a recurrent stress in this ecosystem. However, in contrast to our hypotheses, the biofilm’s functionality was not globally decreasing with a_w_ decrease but showed a contrasting change, from high respiration and hydrolytic enzyme activities under high a_w_, to the increase in the enzyme capacity to degrade lignin together with an increase in prokaryotic biomass as well as bacteriochlorophyll-*a* at the surface sediment (Suppl. Fig. 1). This functional shift linked to enhanced prokaryote biomass occurred at both depths but in a more prominent way at the surface, which may be linked to the more extreme conditions reached (up to 0.7 a_w_). Harsh high salinity and drought conditions may interfere with the activity of hydrolytic enzymes and/or their producers and thus resistant prokaryotes with capacity to degrade lignin or related compounds having aromatic rings (i.e. by phenol oxidase activity) use it to obtain C and nitrogen organic matter sources for their growth. Results suggest that some microbial groups, such as algae and fungi, may resist the harsher conditions by resting states and/or producing resistant structures (EPS, carotenoids), while prokaryotes adapt to the potential organic matter degradation paths either by changing their community or by the growth of resistant bacteria. Probably the interactions between the different groups within the sediment microbial loop are highly responsible for the resistance of the entire microbial community in hypersaline sediments.

## Supplementary Information

Below is the link to the electronic supplementary material.

Supplementary file1 (DOCX 1469 KB)

## Data Availability

No datasets were generated or analysed during the current study.
