# Supplementary material for: Microbial Life in Playa-Lake Sediments: Adapted Structure, Plastic Function to Extreme Water Activity Variations
Source: Microb Ecol. 2024 Nov 9;87(1):137. doi: 10.1007/s00248-024-02454-4 (PMC11550290; doi:10.1007/s00248-024-02454-4)
Supplement: Supplementary file 1 — Supplementary file1 (DOCX 1469 KB) [file 248_2024_2454_MOESM1_ESM.docx]

**SUPPLEMENTARY MATERIAL**


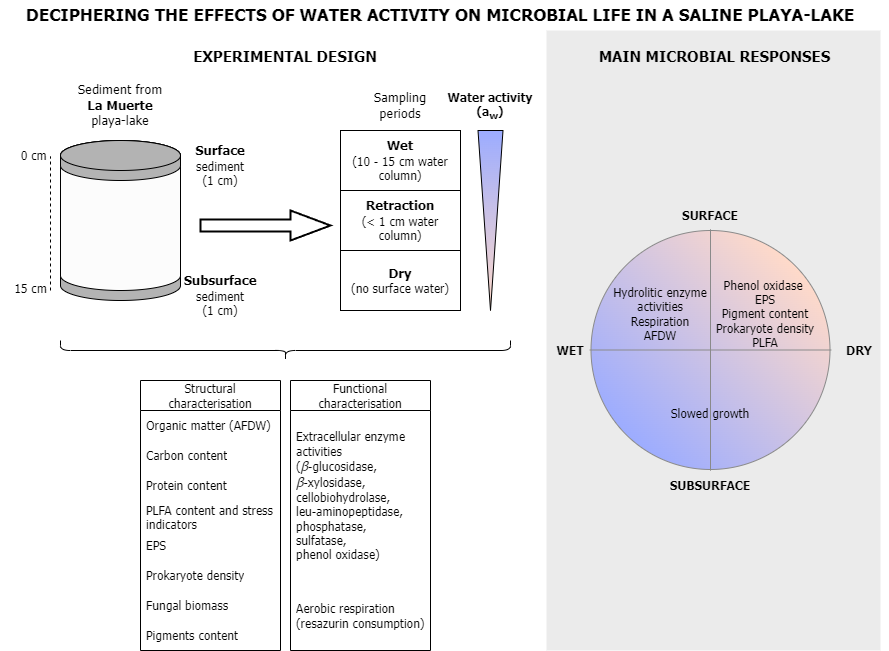


**Supplementary Figure 1.** Schematic diagram summarizing the experimental design (left) and synthesizing the main findings of this study (right).


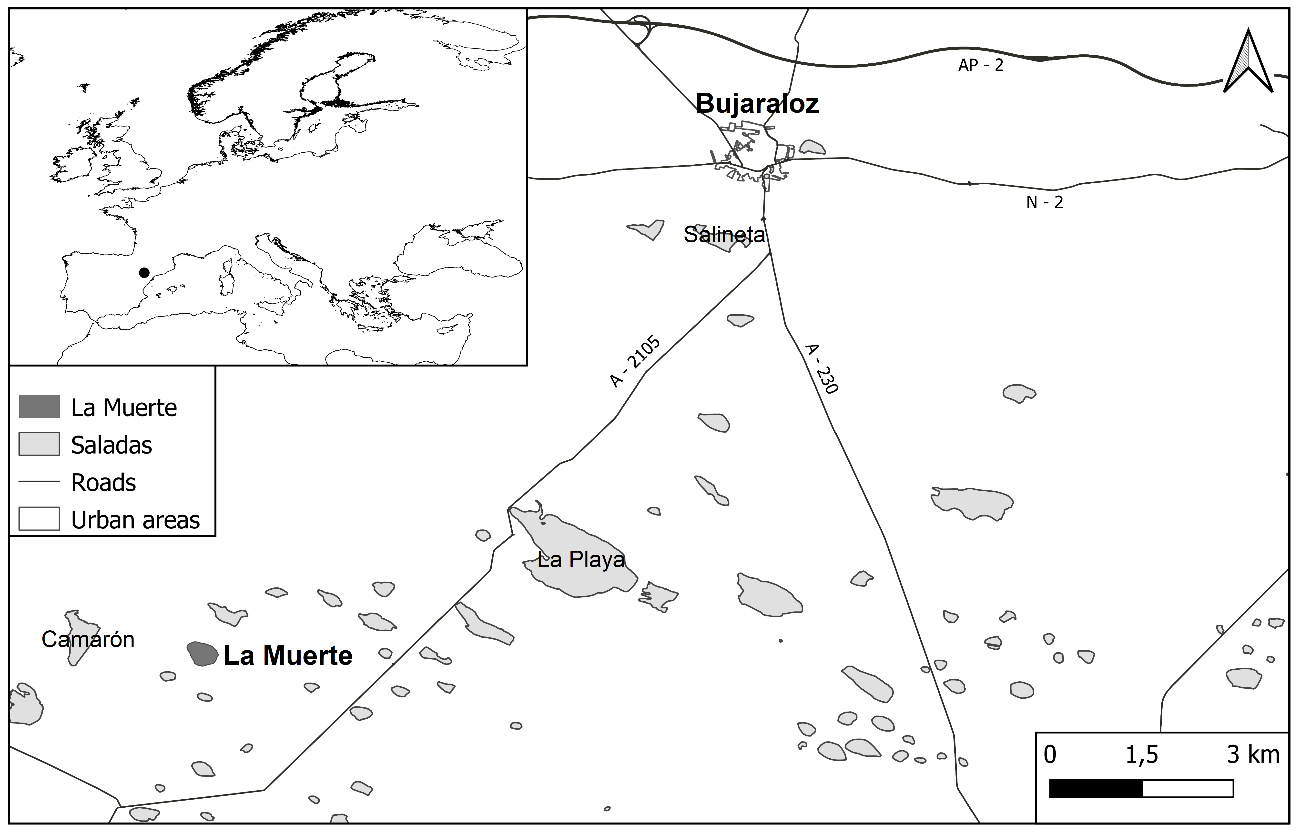


**Supplementary Figure 2.** Map of the location of the study site (La Muerte playa-lake).


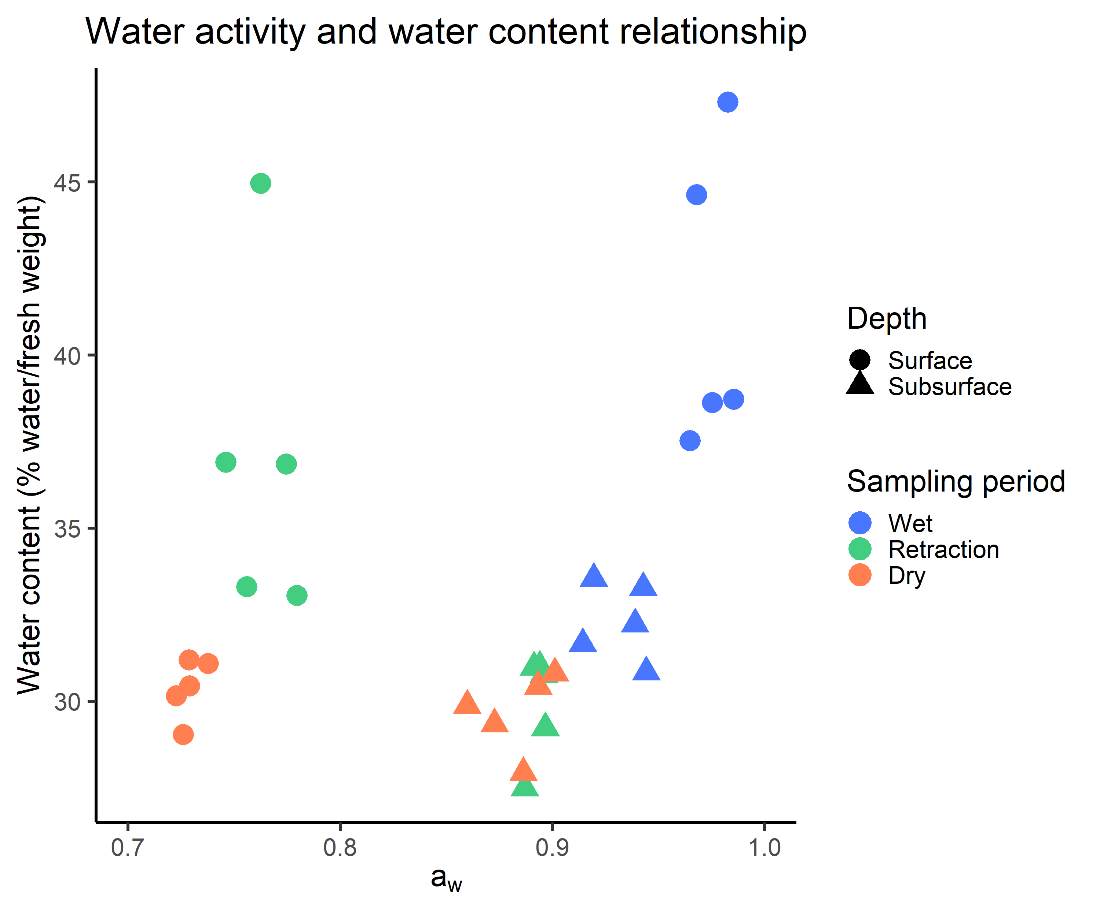


**Supplementary Figure 3.** Relationship between water activity (a_w_) and water content in the hypersaline shallow lake La Muerte. Results include two different sediment depths (surface and subsurface) and three sampling periods (wet, retraction, and dry).


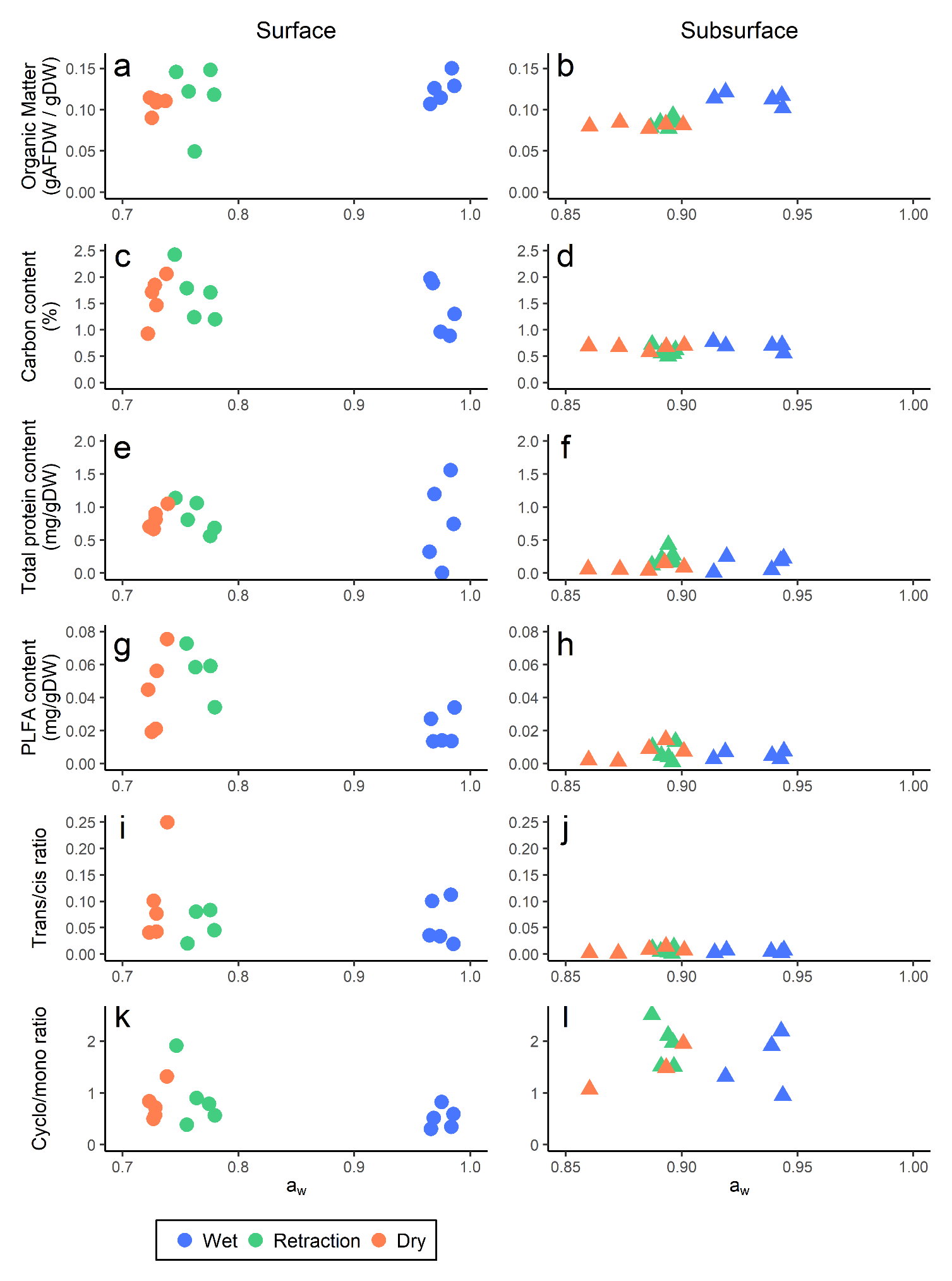

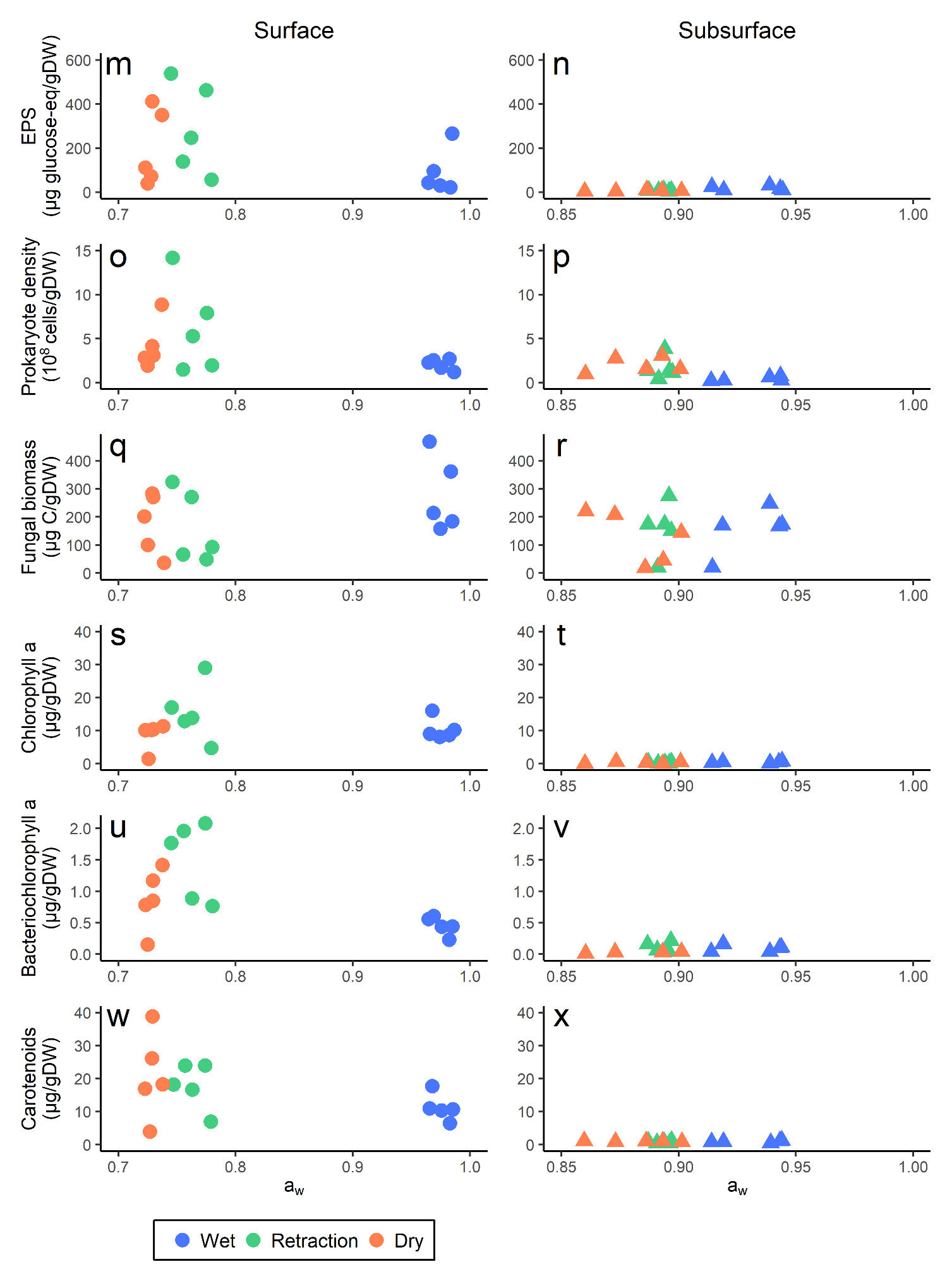


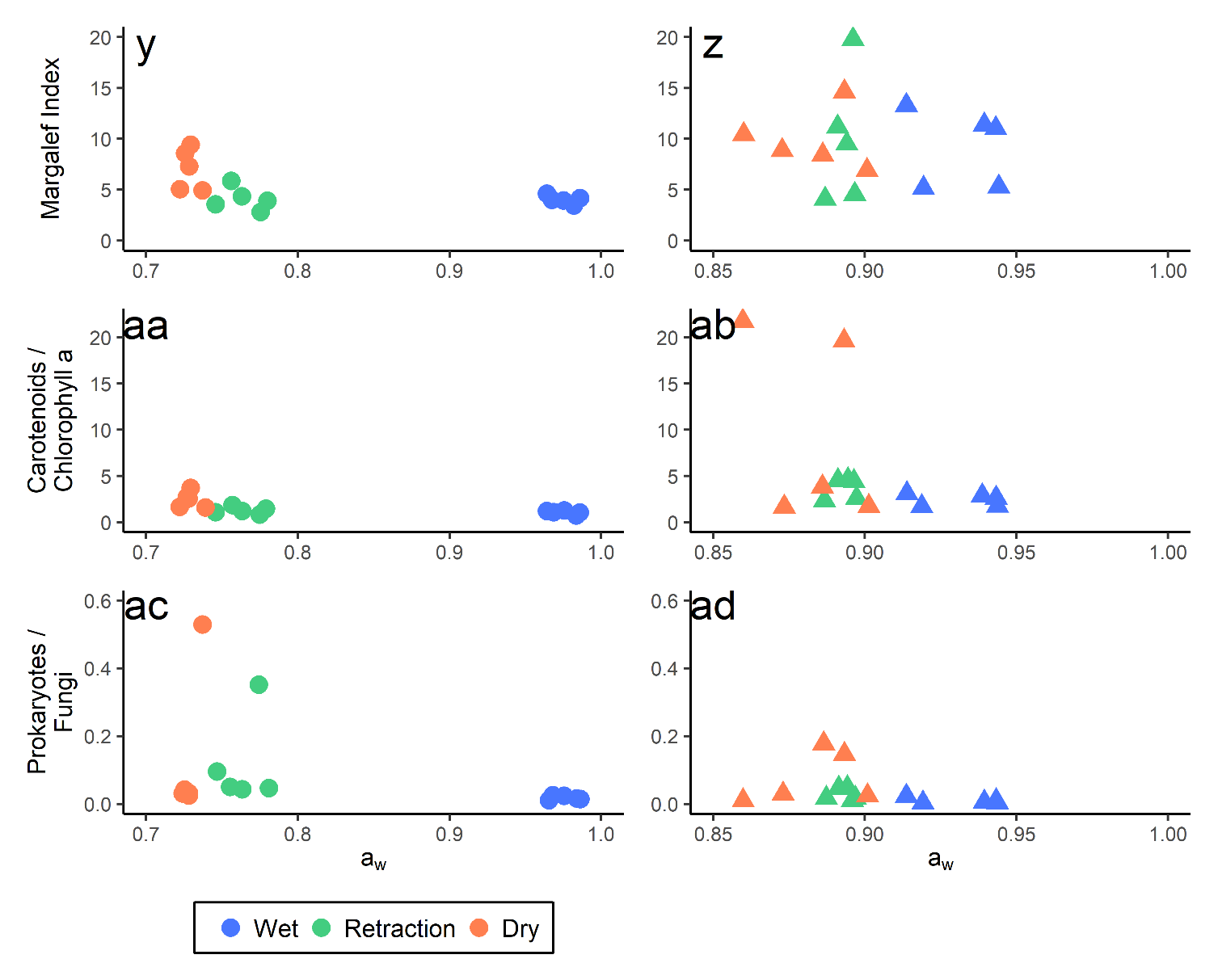


**Supplementary Figure 4.** Structural variables measured in La Muerte for surface (left graphs) and subsurface (right graphs) sediments in the three sampling periods (wet, retraction, and dry). a, b) organic matter; c, d) carbon content; e, f) total protein content; g, h) PLFA content; i, j) trans/cis ratio; k, l) cyclo/mono ratio; m, n) extracellular polymeric substances, EPS; o, p) prokaryote density; q, r) fungal biomass; s, t) chlorophyll *a*; u, v) bacteriochlorophyll *a*; w, x) carotenoids; y, z) Margalef Index; aa, ab) carotenoid/chlorophyll *a*; ac, ad) prokaryotes/fungi.


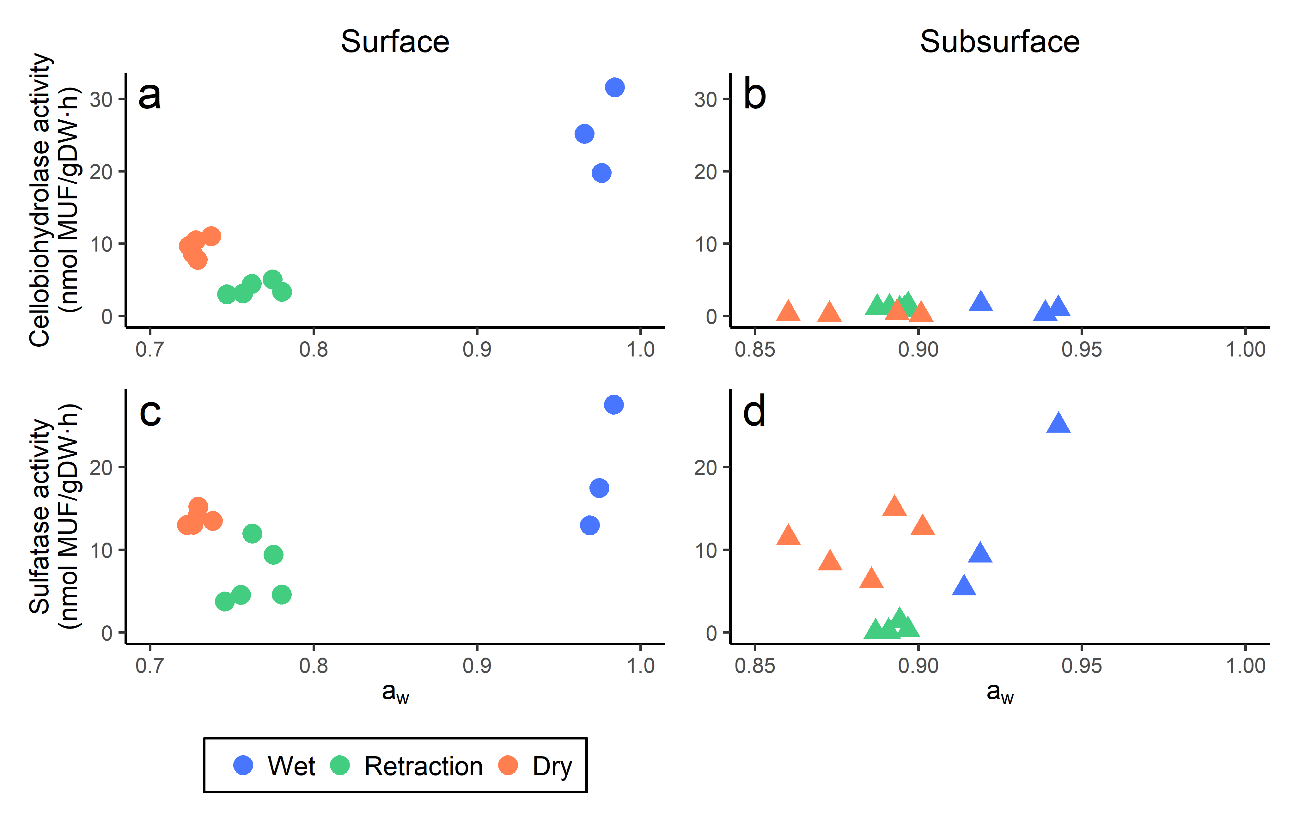


**Supplementary Figure 5.** Extracellular enzyme activities measured in La Muerte for surface (left graphs) and subsurface (right graphs) sediments in the three sampling periods (wet, retraction, and dry). a, b) cellobiohydrolase activity; c, d) sulfatase activity.
